# Capsaicin: Emerging Pharmacological and Therapeutic Insights

**DOI:** 10.3390/cimb46080468

**Published:** 2024-07-24

**Authors:** Elena Madalina Petran, Argyrios Periferakis, Lamprini Troumpata, Aristodemos-Theodoros Periferakis, Andreea-Elena Scheau, Ioana Anca Badarau, Konstantinos Periferakis, Ana Caruntu, Ilinca Savulescu-Fiedler, Romina-Marina Sima, Daniela Calina, Carolina Constantin, Monica Neagu, Constantin Caruntu, Cristian Scheau

**Affiliations:** 1 Department of Biochemistry, The “Carol Davila” University of Medicine and Pharmacy, 050474 Bucharest, Romania; 2 Department of Toxicology, Grigore Alexandrescu Emergency Children’s Hospital, 011743 Bucharest, Romania; 3 Department of Physiology, The “Carol Davila” University of Medicine and Pharmacy, 050474 Bucharest, Romania; 4 Akadimia of Ancient Greek and Traditional Chinese Medicine, 16675 Athens, Greece; 5 Elkyda, Research & Education Centre of Charismatheia, 17675 Athens, Greece; 6 Department of Radiology and Medical Imaging, Fundeni Clinical Institute, 022328 Bucharest, Romania; 7 Pan-Hellenic Organization of Educational Programs (P.O.E.P), 17236 Athens, Greece; 8 Department of Oral and Maxillofacial Surgery, The “Carol Davila” Central Military Emergency Hospital, 010825 Bucharest, Romania; 9 Department of Oral and Maxillofacial Surgery, Faculty of Dental Medicine, “Titu Maiorescu” University, 031593 Bucharest, Romania; 10 Department of Internal Medicine, The “Carol Davila” University of Medicine and Pharmacy, 050474 Bucharest, Romania; 11 Department of Internal Medicine and Cardiology, Coltea Clinical Hospital, 030167 Bucharest, Romania; 12 Department of Obstetrics and Gynecology, The “Carol Davila” University of Medicine and Pharmacy, 020021 Bucharest, Romania; 13 The “Bucur” Maternity, “Saint John” Hospital, 040294 Bucharest, Romania; 14 Department of Clinical Pharmacy, University of Medicine and Pharmacy of Craiova, 200349 Craiova, Romania; 15 Immunology Department, Victor Babes National Institute of Pathology, 050096 Bucharest, Romania; 16 Department of Pathology, Colentina University Hospital, 020125 Bucharest, Romania; 17 Faculty of Biology, University of Bucharest, 76201 Bucharest, Romania; 18 Department of Dermatology, “Prof. N.C. Paulescu” National Institute of Diabetes, Nutrition and Metabolic Diseases, 011233 Bucharest, Romania; 19 Department of Radiology and Medical Imaging, “Foisor” Clinical Hospital of Orthopaedics, Traumatology and Osteoarticular TB, 021382 Bucharest, Romania

**Keywords:** capsaicin, bioavailability, physiopathology, anti-cancer effects, local effects, adverse effects

## Abstract

Capsaicin, the most prominent pungent compound of chilli peppers, has been used in traditional medicine systems for centuries; it already has a number of established clinical and industrial applications. Capsaicin is known to act through the TRPV1 receptor, which exists in various tissues; capsaicin is hepatically metabolised, having a half-life correlated with the method of application. Research on various applications of capsaicin in different formulations is still ongoing. Thus, local capsaicin applications have a pronounced anti-inflammatory effect, while systemic applications have a multitude of different effects because their increased lipophilic character ensures their augmented bioavailability. Furthermore, various teams have documented capsaicin’s anti-cancer effects, proven both in vivo and in vitro designs. A notable constraint in the therapeutic effects of capsaicin is its increased toxicity, especially in sensitive tissues. Regarding the traditional applications of capsaicin, apart from all the effects recorded as medicinal effects, the application of capsaicin in acupuncture points has been demonstrated to be effective and the combination of acupuncture and capsaicin warrants further research. Finally, capsaicin has demonstrated antimicrobial effects, which can supplement its anti-inflammatory and anti-carcinogenic actions.

## 1. Introduction

The most prominent pungent principle in the hot peppers (*Capsicum annuum*) of the genus Capsicum [1] is capsaicin (8-methyl-*N*-vanillyl-6-nonenamide), an organic nitrogenous compound within the lipid group [2]. It must be noted that the name capsaicin was originally used to refer to a multitude of substances originally isolated from *C. oleoresin*; these compounds are now known as capsaicinoids [3], a distinction made after the 1960s [4].

Interestingly, it has been discovered that the cultivation of chilli peppers began around the 5th millennium BC [5], thus rendering them amongst the oldest cultivated plants; their origin is estimated to be somewhere in Bolivia [6]. Chilli peppers came to Europe only after the discovery of the New World and the subsequent Columbian Exchange, which had far-reaching consequences [7]; this is hardly surprising as numerous foodstuffs followed this historical process [8,9,10]. Subsequently, chilli peppers were swiftly adopted by many cultures and, as such, are ingredients in many local and traditional dishes [11]. It is believed that the synthesis of capsaicin within the plant is part of a defence mechanism developed against consumption by herbivores and micro-organism infestations [12]; however, not all chillies are pungent [13].

Extracted initially as an impure formulation by C.F. Bucholz (1770–1818), it was termed “capsicin” [14,15]. The original compound, isolated almost completely in 1876 by J.C. Thresh (1850–1932) [16,17,18], was a colourless substance of a crystalline structure—though purified in the 1870s, the first description of its structure is recorded in 1919 [19]—this is not surprising given that the complete isolation of the compound was achieved only in 1898 by K. Micko [20,21]. Based on the original isolation of capsaicin and the identification of its chemical and physical properties, capsaicinoids, of which capsaicin is a member, are defined as chemical compounds which have similar structures and properties as capsaicin [22]. 

Regarding capsaicin in particular, in its pure form, it is a solid, colourless, hydrophobic, highly volatile and highly pungent substance [23]; if heated to decomposition (80–140 °C), the fumes emitted are toxic nitrogen oxides [24]. Its chemical formula is C_18_H_27_NO_3_ [25]. Capsaicin naturally occurs in its trans form, although a cis-isomer also exists [26].

The biosynthetic path of capsaicin, as described in research [27], involves a chemical reaction between vanillylamine and 7-methyloct-5-ene-1-carboxylic acid chloride; this reaction takes place in the fleshy parts of the fruits. In the seeds of these fruits, no capsaicin is produced; however, the white part of their inner wall contains the highest capsaicin concentration, and it is where the seeds are attached [26]. It is interesting to note that under stress conditions, the capsaicin production of the plant increases [28,29]. Currently, artificial synthesis of capsaicin is possible using a number of different methods [30]. The first artificial synthesis of capsaicin was recorded in 1930 [31]; a number of methods have been developed lately to enhance its production [32,33,34,35], given its high demand for research and applications.

The most oft-used scale to measure capsaicin’s, or indeed any other compound’s, pungency is the Scoville Heat Unit (SHU) scale, proposed in the early 1990s [36]; this is based on the subjective pungency perception of people consuming pungent substances and foods. It is a linear scale, and it can exceed even 10^6^ SHU for the hottest peppers containing the highest amounts of capsaicinoids [37].

Due to its properties, capsaicin has a number of already established clinical and industrial uses (Table 1), while a number of novel clinical applications are under discussion. Outside of medical applications, the very potent irritative effect of capsaicin on mucosae [38] means that it constitutes an important component in pepper spray products [39,40,41,42].

In this review, we will present a comprehensive analysis of the pharmacodynamics and pharmacokinetics of capsaicin and elaborate on its pharmacotoxicity. Given that our study focuses on the pharmacological properties of capsaicin, the most prominent local, systemic and anticarcinogenic applications of capsaicin will be presented in detail. The applications of capsaicin in traditional medicine will also be addressed, and current evidence of the most promising avenues of future research will be reported.

## 2. Materials and Methods

This review was conducted by systematically searching major electronic databases, including PubMed, Scopus, and Web of Science. The search strategy was developed using a combination of Medical Subject Headings (MeSH) and keywords. MeSH terms included “Capsaicin”, “Biochemical Properties”, “Therapeutic Applications”, and “Pharmacology”. Keywords related to these terms, such as “pain management”, “analgesic effect”, and “TRPV1 receptor”, were also used to ensure comprehensive coverage. 

Inclusion Criteria: Peer-reviewed articles and studies focused on the biochemical properties, therapeutic applications, or pharmacological insights of capsaicin. 

Exclusion Criteria: Studies not specifically addressing capsaicin, non-peer-reviewed literature, such as abstracts, conference proceedings, and grey literature.

The search strategy aimed to capture a wide array of relevant studies to provide an updated and comprehensive overview of capsaicin. Each database was searched using tailored strategies to maximise the retrieval of pertinent studies. The selection process involved screening titles and abstracts, followed by a full-text review to ascertain eligibility based on the predefined inclusion and exclusion criteria.

## 3. Pharmacokinetics and Pharmacodynamics of Capsaicin

Being liposoluble, capsaicin is well absorbed orally, as well as at the digestive tract level; internal administration means that it will also reach systemic circulation, while systemic administration itself is also possible [76]. The absorption of capsaicin takes place at the level of the stomach and the intestine, varying between 50% and 90%; at any rate, it is invariably a passive process [77]. The intestinal epithelial cells can metabolise a small percentage of the absorbed capsaicin [78]. Despite its lipophilicity, which correlates with good skin absorption [79], capsaicin does not reach sufficiently high levels in the plasma following local or transdermal administration to exert its effects systemically [80].

Following its internal (oral) administration, capsaicin is hepatically metabolised [78], with the small aforementioned exception. Based on in vitro studies, it has been established that, following its rapid metabolisation, the three most important metabolites are 16-hydroxycapsaicin, 17-hydroxycapsaicin, and 16,17-hydroxycapsaicin; vanillin is a minor metabolite [81,82]. Based on a subsequent study [83], another metabolite of capsaicin was identified, which corresponds to a compound occurring after phase I demethylation and dehydrogenation. A glycine and a glutathione conjugate were also identified. At any rate, a small percentage of capsaicin is fecally excreted, while most of the elimination is renal for the glucuronide metabolites of capsaicin [84]. 

While it may be assumed that cytochrome P_450_ enzymes are, most probably, involved in capsaicin metabolism [85,86], in human skin cell studies, the biotransformation process has been found to be slow, and most of the administered capsaicin did not undergo any changes [87]—this last fact has important implications for medicinal capsaicin applications. The half-life of capsaicin in the human body was determined to be 25 min [87]; conversely, the local application of a 3% capsaicin solution yielded a value of 24 h [88,89]. More recent research [90] has identified a novel metabolic pathway in the human body, resulting in macrocyclic diene and imide metabolites.

The first physiological action of capsaicin is observed when it binds to the TRPV1 (transient receptor potential cation channel subfamily V member 1) [91]; capsaicin is a potent TRPV1 receptor agonist [92]. Such is the physiological importance of this receptor, and of temperature and mechanically activated channels in general, that research on them resulted in the awarding of the 2021 Nobel Prize in Physiology or Medicine to David Julius and Ardem Patapoutian [93,94,95] based on their previous research (e.g., [96,97,98,99,100,101]).

This receptor, which is also called capsaicin or vanilloid receptor 1 [102], can be activated, apart from its agonists, by a temperature higher than 43 °C and a pH lower than 5.2. Some examples of endogenous agonists are bradykinin and prostaglandins [103]. The receptor function is associated either with protein kinase A or protein kinase C [104,105]. The activation of this receptor enables it to exert its modulatory activity; its principal role is body temperature regulation [106,107]. The heat perception properties of TRPV1 have also been proposed by Tominaga et al. [108] who have also noted that TRPV1 is instrumental in peripheral nociception. The nerve signals resulting from its activation reach all up to the spinal cord and eventually the brain.

TRPV1 was identified in the central nervous system as well as in the sensory neurons of the dorsal root ganglion [109]. At the level of the cardiovascular system, it can also be found in vascular smooth muscle cells and endothelial cells [110]; of course, neural TRPV1 activation will also have cardiovascular-related effects [111]. It must be noted that TRPV1 does not seem to be expressed in cardiomyocytes [112], but there is a report indicating that it is possibly expressed in the nerve fibres of the epicardium [113]. At the level of the respiratory system, TRPV1 is found in the airway epithelial cells and in the T cells of the upper and lower airways [114]; interestingly, the expression of TRPV1 in the respiratory system seems to vary in different pathological situations [115,116]. At the level of the gastrointestinal tract (GIT), TRPV1 can be found in the submucosal nerve plexus, myenteric nerve plexus, gastrointestinal mucosal cells, parietal and antral G cells [117].

At the level of the integumentary system, TRPV1 can be found in a number of different cell types, namely unmyelinated type C and thin myelinated A_δ_ sensory nerve fibres, keratinocytes, sebocytes, dermal blood vessels, mast cells, fibroblasts, hair follicles, and vascular smooth muscle cells [118,119,120]. In the eyes, TRPV1 is present in corneal cells [121] and retinal ganglion cells [122].

Capsaicin induces a variety of TRPV1-mediated sensations with different intensities, from warming and tingling up to burning [123,124]. Another aspect that must be considered is that capsaicin-induced activation of TRPV1 is more persistent compared to the effect of other natural agonists. In fact, capsaicin is a more potent agonist compared to any endogenous TRPV1 agonists—which have been analysed in detail in recent studies [125]—and, although being the most characteristic exogenous TRPV1 agonist [126], there are some more potent such agonists, like resiniferatoxin [127,128] and a number of recently researched compounds [129]. The capsaicin-induced activation of TRPV1 is associated, at least in a number of cases, with a relative desensitisation [130].

Capsaicin exerts a host of different effects at cellular [131,132,133,134,135] and subcellular levels [136,137]. Two pathways are thought to exist via which capsaicin may inhibit nociception: a TRPV1-dependent one and a TRPV1-independent one. The TRPV1-independent effects are associated with changes in the lipid membrane properties, the modulation of voltage-gated ion channels and direct binding to other enzymes and transporters [138,139,140]. The TRPV-1-dependent pathway implies activation of the receptor and subsequent desensitisation, which can be modulated by various factors, including cAMP/PKA-dependent activation [141,142]. Both the dependent and independent effects are most possibly associated with the reduced nociception caused by capsaicin [138].

**Figure 1:**
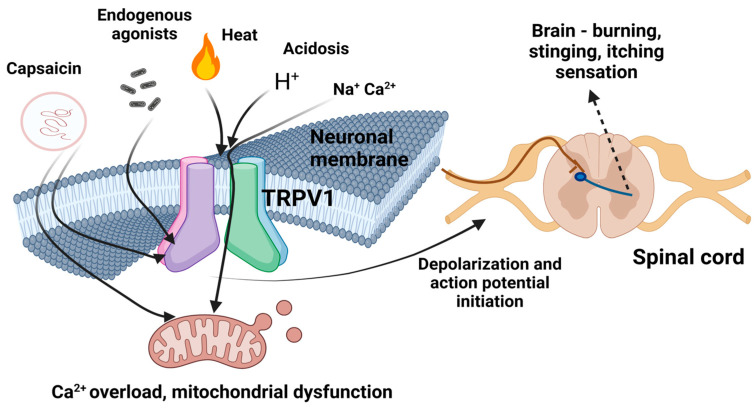
Capsaicin activation of TRPV1. Capsaicin induces sensory neuronal depolarization and local sensitisation to activation by heat, acidosis, and endogenous agonists. Topical application of capsaicin induces sensations of heat, burning, stinging, or itching. When high concentrations of capsaicin are used, or there are repeated applications in cutaneous nociceptors, a de-functionalization process is induced.

The aforementioned blockade of nociceptors, when coupled with the capacity of reducing the inflammation-associated substance P [143], renders capsaicin a good candidate for a non-narcotic analgesic [144,145]; indeed, the new technologies available render the design of pharmacological capsaicin analogues a possible and potent eventuality [126]. In the manifestation of analgesic effects, the indirect blockage of voltage-gated Na^+^ channels may also play a role [146,147]. In addition, some other associated capsaicin-induced actions comprise the degeneration of epidermal nerve fibres after prolonged local administration [148]. A number of researchers have presented the most recent developments regarding the novel analgesic capsaicin applications [149,150,151,152]. A general outline of the TRPV1-mediated activation by capsaicin is presented in Figure 1.

## 4. Indications and Therapeutical Uses of Capsaicin

In general, the uses of capsaicin are numerous and varied, ranging from medicine, either human or veterinary, to uses in agriculture, the food industry, and fragrances. In human medicine, we distinguish between local and systemic applications (Table 2 and Table 3).

### 4.1. Capsaicin as a Local Agent

As discussed above, capsaicin is lipophilic and can hence be absorbed readily, reaching and activating the TRPV1 receptor, which can be found both in nociceptive and non-nociceptive structures. The binding of capsaicin leads to receptor activation, upon which a prolonged desensitisation state prevails; this second state renders the use of capsaicin very promising in chronic pain syndromes, as well as against hyperplasias, inflammation and inflammatory skin diseases, various dermatoses, as well as chemotherapy-induced and radiotherapy-induced mucositis [153].

For local applications, a variety of capsaicin preparations are available, such as creams, gels, liquids and patches [154], while novel formulations comprise nanolipid carriers [155,156,157], flexible membrane vesicles [158] and alginate microcapsules [159]. These last formulations can be considered better in that they improve the pain threshold in a dose-dependent manner, compared to the older locally-administered drugs; the positive effect is exerted through the reduction of tissue prostaglandin E2 levels, while skin irritation is also reduced [160]. The most prominent local capsaicin applications are presented in Table 2.

**Table 2:** Local applications of capsaicin.

Indication	Formulation	Effect	Action Mechanism	Type of Study	Year	References
Neuralgia associated with herpes zoster infection	Cream 0.025% 3–4 times/day for 2 days	Antalgic	Substance P depletion/prevention of reuptake	In vivo—human	1988	[161]
Neuralgia-associated periocular and facial pain	15 mg of capsaicin cream 2 daily	Antalgic	Substance P depletion	In vivo—human	1988	[162]
Facial apocrine chromhidrosis	Cream 1–2 times/day	Antalgic and possible vasodilation inhibition	Substance P depletion/prevention of reuptake	In vivo—human	1989	[163]
Reflex sympathetic dystrophy	Cream 0.025% 1–2 times/day for 3 weeks	Antalgic	Substance P depletion	In vivo—human	1990	[164]
Diabetic neuropathy	0.075% capsaicin cream for 8 weeks	Antalgic	Substance P depletion/desensitization of C nociceptal fibers	In vivo—human	1991	[165]
Chronic severe painful diabetic neuropathy unresponsive or intolerant to conventional therapy.	Cream 0.075% 4 times/day for 8 weeks	Antalgic	Substance P depletion/desensitization of warm nociceptors, polymodal nociceptors and nociceptive afferents	In vivo—human	1992	[166]
Osteoarthritis	Cream 0.075%	Antalgic	Unknown	In vivo—human	1992	[167]
Postmastectomy pain syndrome	0.075% capsaicin 4–5 times/day for 4–6 weeks	Antalgic	Unknown	In vivo—human	1992	[57]
Notalgia paresthetica	Cream 0.025 percent for four months	Antalgic, antipruritic	Uncertain	In vivo—human	1992	[168]
Haemodialysis-induced pruritus	Cream 0.025% 4 times/day for 6 weeks	Anti-pruritic	Substance P prevention of reuptake/depletion/desensitization of unmyelinated c fibers of cutaneous nerves	In vivo—human	1992	[67]
Chronic postherpetic neuralgia	0.075% cream	Antalgic	Possible desensitisation of nociceptors	In vivo—human	1993	[169]
Pruritic psoriasis	0.025% cream 4 times/day for 6 weeks	Antipruritic	Substance P depletion	In vivo—human	1993	[170]
Post-mastectomy pain syndrome	0.025% cream 3 times/day for 2 months.	Antalgic	Substance P depletion	In vivo—human	1993	[171]
Cluster headache	Intranasal 3% camphor in 0.025% capsaicin cream for 7 days	Antalgic	Substance P depletion	In vivo—human	1993	[172]
Aquagenic pruritus	Cream 0.025%, 0.5% or 1.0% 3 times/day for 4 weeks	Antipruritic	Substance P depletion	In vivo—human	1994	[173]
Erythromelalgia	Cream 0.025% every 12 h for 2 months	Antalgic	Substance P depletion	In vivo—human	1994	[174]
Trigeminal neuralgia manifesting as intraoral pain	Cream 0.025% 4 times/day for 4 weeks	Antalgic	Substance P depletion/desensitization of c nociceptors	In vivo—human	1994	[175]
Chronic neck pain	Cream 0.025% 4 times/day for 5 weeks	Antalgic	Substance P depletion	In vivo—human	1995	[176]
Meralgia paraesthetica	Cream 0.025% 5 times/day for 15 days	Antalgic	Substance P depletion/prevention of reuptake/desensitisation of C-polymodal nociceptors	In vivo—human	1995	[177]
Skin flap survival	Silicongel 0.025%	Increased flap survival	platelet disaggregation	In vivo—animal	1996	[178]
Haemodialysis-induced pruritus	Cream 0.025% 4 times/day	Antipruritic	Substance P depletion	In vivo—human	1996	[68]
Herpes zoster ophthalmicus neuralgia	Cream 0.025% 5 times/day for 4 weeks	Antalgic	Substance P depletion	In vivo—human	1997	[179]
Complex regional pain syndromes and neuropathic pain	Cream 7.5%	Antalgic	Desensitization of C-fiber nociceptors	In vivo—human	1998	[180]
Atopic eczema	Cream 0.05% 3 times/day for 5 days	Antipruritic	Substance P depletion/inhibition	In vivo—human	1998	[181]
Diffuse eosinophilic sinonasal polyposis	3 days 0.5 mL 30 micromol/L capsaicin solution and on days 4 and 5, 100 micromol/L	Improved subjective and endoscopy scores	Possible neurotoxic effect	In vivo—human	2000	[182]
Pain of osteoarthritis	0.025% capsaicin, 1.33% glyceryl trinitrate (one part 0.075% capsaicin, two parts 2% glyceryl trinitrate)	Antalgic	Nociceptive blocking/increase in perfusion of glyceryl trinitrate	In vivo—human	2000	[58]
Pain following spinal cord injury	Cream 0.025% 4 times/day	Antalgic	Substance P depletion/desensitization of unmyelinated afferent C fibers	In vivo—human	2000	[183]
Prurigo nodularis	Cream 0.025% to 0.3% 4 to 6 times daily for 2 weeks up to 10 months	Antipruritic	Substance P depletion	In vivo—human	2001	[184]
Complex regional painsyndrome type I	Cream 0.075% 2 times/day for 6 weeks	Antalgic	Desensitization of epidermal C fibers	In vivo—human	2001	[185]
Atopic dermatitis	Lotion 0.025% 2 times/day for 6 weeks	Antipruritic	Possible desensitization or neuroinhibition	In vivo—animal	2002	[186]
Abdominal wall scar pain	Cream 0.075% 3 times/day usually for 2 weeks and after that 2 times/day	Antalgic	Desensitization of vanilloid subtype 1 (VR1) receptors	In vivo—human	2002	[187]
Post-operative nausea and vomiting after abdominal hysterectomy	Capsicum plaster with 345.80 mg of powdered capsicum for at least 30 min before anesthesia and eight hours after surgery on the acupuncture point P6 or the Korean acupuncture point K-D2	Antiemetic	Desensitization of K-D2 hand point zone	In vivo—human	2002	[64]
Haemodialysis related pruritus	Cream 0.05% liniment 3 times/day for 5 days	Antipruritic	Substance P depletion/desensitization of epidermal nerve fibers	In vivo—human	2003	[188]
Meningeal nociception and headache	10 μM topical	Antalgic	Desensitization of afferent fibers	In vivo—animal	2003	[189]
Saphenous neuralgia	Cream 5 times/day for 2 months	Antalgic	Substance P depletion	In vivo—human	2003	[190]
Idiopathic intractable pruritus ani	Capsaicin ointment 0.006–0.012% (depending on dilution) for 4 weeks followed by a week washout and by 4 weeks of placebo (menthol 1%)	Antipruritic	Substance P depletion	In vivo—human	2003	[69]
Detrusor hyperreflexia	10 mM topical for 3 months intravesical instillations	Improving continence and bladder function	Possible desensitization of sensory Aδ and unmyelined C fibers	In vivo—human	2004	[191]
Burning mouth syndrome	Oral capsaicin 0.25% 3 times/day for 1 month	Antalgic	Desensitisation of type-C pain receptors	In vivo—human	2004	[192]
Detrusor hyper-reflexia in spinal cord-injured patients	Intravesical instillation of 1 mmol/L CAP diluted in glucidic solvent for 3 months	Improving continence and bladder function	Desensitization or blocking of afferent C-nerve fibres	In vivo—human	2004	[73]
Prevention of post-operative sore throat	Capsicum plaster with powdered capsicum 345.8 mg on the Korean acupuncture point K-A20	Antalgic	Unknown—presumably release of endogenous opioids	In vivo—human	2004	[66]
Post-operative nausea and vomiting after anaesthesia in middle ear surgery	Capsicum plaster withcapsicum oleoresin 1% *w*/*w* on acupuncture point P6	Antiemetic	Release of endogenous opioids/modulation of neurotransmitters of the vestibular system	In vivo—human	2005	[65]
Post-operative nausea and vomiting after laparoscopic cholecystectomy	Capsaicin ointment with oleoresin capsicum equivalent to capsaicin 0.075% *w*/*w* and methyl salicylate I.P. 20% *w*/*w* on the Korean acupuncture point K-D2	Antiemetic	Blocking of synthesis of substance-P from sensory C-fibers/desensitisation of afferent sensory nerves.	In vivo—human	2005	[63]
Acute lobular panniculitis	0.075% capsaicin cream 5 times/day for 3 weeks	Antalgic, antithrombotic	Substance P depletion	In vivo—human	2005	[193]
Acute lipodermatosclerosis	0.075% capsaicin cream for 1–2 weeks followed by a month of continuation	Antalgic, antithrombotic	Substance P depletion	In vivo—human	2005	
Post-abdominal hysterctomy pain	Plaster of capsaicin (0.046% *w*/*w*) mixture of powdered capsicum 345.80 mg and capsicum tincture 34.58 mg at ST36 acupuncture point	Antalgic, antiemetic	Release of endogenous opioids (possibly)	In vivo—human	2006	[194]
Skinmorphological changesin patients with growth hormone deficiency and in the elderly	Cream of 0.01% capsaicinoids (dihydrocapsaicin and nordihydrocapsaicin)/0.01% capsinoids (capsiate, dihydrocapsiate and nordihydrocapsiate)	Increased skin elasticity	Increased dermal IGF-I levels	In vivo—human	2007	[195]
Painful HIV-associated distal sensory polyneuropathy (DSP)	Patch 640 microg/cm^2^, 8% *w*/*w* 60 min 1 time/day for 12 weeks	Antalgic	Desensitization of cutaneous nociceptors	In vivo—human	2008	[196]
Post-operative pain after orthognathic surgery	Capsicum plaster with 345.80 mg powdered capsicum and 34.58 mg capsicum tincture applied on LI4 acupuncture point	Antalgic, antiemetic	Blocking of transport and synthesis of substance P from sensory C-fibers	In vivo—human	2009	[197]
Migraine pain	Capsaicin jelly with 0.1% capsaicin	Relief and prevention of mild migraines	Substance P depletion	In vivo—human	2010	[198]
Chronic soft tissue pain	0.05% capsaicin cream	Antalgic	Substance P depletion/degeneration of epidermal nerve fibres	In vivo—human	2010	[199]
Haemodialysis-induced uremic pruritus	0.03% capsaicin ointment 4 times/day for 4 weeks	Antipruritic	Substance P depletion	In vivo—human	2010	[70]
Cardiac ischemia	5 mL of 0.1% capsaicin cream applied to abdomen; experimental conditions different per animal group	Remote cardioprotective	Release of blood-bornecardioprotective factors	In vivo—animal	2012	[200]
Trigeminal Postherpetic neuralgia	Capsaicin 8% patch; single 60 min application	Antalgic	Substance P depletion/defunctionalization of TRPV1 receptors on sensory nerve endings	In vivo—human	2012	[201]
Visceral obesity	0.075% capsaicin cream for 7 + 7 weeks (pretreatment and post-treatment)	Antiinflammatory, antilipidemic, anti-diabetic	Increased adiponectin, PPARα, PPARγ, visfatin, adipsin and decreased TNF-α and IL-6	In vivo—animal	2013	[202]
Fibromyalgia	0.075% capsaicin cream 3 times/day for 6 weeks	Antalgic	Substance P depletion/desensitization of polymodal nociceptors	In vivo—human	2013	[203]
Peripheral neuropathic pain	8% capsaicin cutaneous patch for 30 min to the feet and 60 min to other parts of the body	Antalgic	Probably substance P related	In vivo—human	2014	[204]
Posttraumatic neuropathic pain	8% capsaicin cutaneous patch for 30 min for the feet and 60 min for other locations every 90 days	Antalgic, anti-inflammatory	Defunctionalisation of nociceptors	In vivo—human	2014	[205]
Arthritis and associated inflammo-musculoskeletal disorders	Topical ethosomal capsaicin	Antalgic, anti-inflammatory	Substance P inhibition	In vivo—animal	2015	[206]
Post-herpetic neuralgia	Liposomal non-ionic capsaicin cream (0.025%) 2–3 times/day for 6 weeks followed by a 2-week cessation	Antalgic	Unclear	In vivo—human	2015	[207]
Intraoral somatosensory sensitivity	30 μL of 5% capsaicin on a paper disc for 15 min	Mechanical desensitization	Desensitization of C-nociceptors	In vivo—human	2015	[208]
Lichen amyloidosis	8% capsaicin patch with 179 mg capsaicin for 60 min	Antipruritic	Defunctionalization of transient receptor potential ion channel vanilloid-1	In vivo—human	2016	[209]
Cannabinoid hyperemesis syndrome	Capsaicin cream	Antiemetic	Substance P depletion	In vivo—human	2017	[210]
Burning mouth syndrome	0.01% or 0.025% oral capsaicin gel 3 times/day for 14 days	Antalgic	Substance P depletion/desensitization of transient receptor potential ion channel vanilloid-1	In vivo—human	2017	[211]
Neuropathic pain caused by lumbosacral radiculopathies	8% capsaicin patch for 30 min for the feet and 60 min for other locations	Antalgic	Desensitization of lumbosacral spinal nerves	In vivo—human	2017	[212]
Histamine-induced pruritus on canine skin	3 mL of 0.1% capsaicin solution 2 times/day for 8 days	Antipruritic	Desensitization of the sensory afferents	In vivo—animal	2018	[213]
Neurogenic inflammation	Topical 50 μM of capsaicin for 15 min after the topical application of 200 μM of capsazepine	Neutrophil leukocyte activation	Increased leukocyte rolling and adhesion, increased expression of E-selectin and ICAM-1	In vivo—animal	2018	[214]
Myofascial pain syndrome	10 g capsaicin cream 8%, for 30 min	Antalgic	Substance P depletion/inhibition (probably)	In vivo—human	2019	[215]
Acute musculoskeletal injuries	Capsaicin gel of 0.05% capsaicin 3 times/day for 72 h	Antalgic	Substance P depletion/inhibition (probable)	In vivo—human	2020	[216]
Hepatic staetosis, obesity, dislipidemia and high blood pressure associated with hypoestrogenism	0.75 g/kg capsaicin cream	Anti-obesity, antilipidemic, antihypertensive	Activation of TRPV1 receptors in neurons of the digestive tract/ increased lipid mobilization and oxidation/reduced cholesterol shynthesis	In vivo—animal	2020	[217]
Type 2 diabetic patients with painful peripheral neuropathy	0.075% capsaicin gel	Antalgic	Substance P depletion/defunctionalization of the C fiber nociceptors	In vivo—human	2020	[218]
Trigeminal neuropathic pain	10 µg in 20 µL of vehicle subcutaneously injected	Antalgic	Capsaicin-induced ablation of TRPV1+ afferent terminals	In vivo—animal	2020	[219]
Psoriasis	10 μg of Capzasin-HP cream (0.1% capsaicin) for 2 times/day for 8 days	Anti-inflammatory	Desensitization of TRPV1 nerves/denervation-induced inhibition of cutaneous inflammatory responses	In vivo—animal	2021	[220]
Sensory neuropathic cough	Spray of capsaicin 0.02% to 0.04% for 4 times/day for 2 weeks	Antitussive	Substance P depletion/defunctionalization of thermal, mechanical, chemical, and other sensory nerve endings	In vivo—human	2021	[221]
Cannabinoid-induced hyperemesis syndrome	Capsaicin cream 0.025%	Anti-emetic effect	Substance P depletion/defunctionalization of TRPV1	In vivo—human	2021	[222]
UVB-induced cutaneous hyperalgesia	8% transdermal patch or two vehicle patches	Antalgic	Substance P depletion/defunctionalisation of local nociceptors	In vivo—human	2021	[223]
Idiopathic rhinitis	Nasal spray of 0.01 mM capsaicin	Reduction of nasal symptoms	Substance P depletion	In vivo—human	2021	[224]
Hamartoma tumour syndrome	Patch 8%	Pain relief	Substance P depletion/inhibition	In vivo—human	2022	[225]
Acute trauma pain	0.05% capsaicin gel for 3 times/day for 72 h after discharge from the hospital	Antalgic	Substance P depletion/inhibition (probable)	In vivo—human	2022	[226]
Improved dermal blood flow	Cream 8%	Improved skin oxigenation	Local vasodilation induced by TRPV1-mediated release of substance P, CGRP, and other vasoactive peptides	In vivo—human	2022	[227]
Chronic postsurgical pain	8% capsaicin patch every 3 months	Antalgic	Defunctionalization of transient receptor potential vanilloid-1 (probable)	In vivo—human	2022	[228]
Pain during microfocused ultrasound with visualization (MFU-V) treatment	0.025% capsaicin gel	Antalgic	Defunctionalization of transient receptor potential vanilloid-1	In vivo—human	2023	[151]
Peripheral neuropathic pain	One topical high-concentration capsaicin application	Antalgic	Axon reflex vasodilatation associated with pain reduction	In vivo—human	2023	[229]

Most, if not all, of the local applications mentioned in Table 2 can be combined with anti-inflammatory drugs; this enables augmentation of their effects, thus leading to dose reduction, which diminishes their systemic side effects [230]. It may be observed that an abundance of these applications is associated with the inhibition and/or depletion of substance P; substance P, a bioactive peptide of the tachykinin family [231], is secreted by nerve cells and a host of inflammatory cells [232]. Substance P is associated with neurogenic inflammation [233,234] both systemically and at the level of the skin [235,236,237], the cardiovascular system [238,239], the respiratory [240,241,242], gastrointestinal [243,244,245] and genitourinary [246,247] tracts and also in the cerebral arteries [248,249]. The relative ubiquity of substance P in the human body renders it a prime target for pharmacological interventions in inflammatory diseases [250]. Notably, substance P is associated with infection-induced inflammation, a fact proven in both human and animal models [251,252,253,254]; taking into account the already proven antimicrobial properties of capsaicin, this could prove an interesting research avenue. In an experimental setting, capsaicin has also been used locally to demonstrate the effects of psychological triggers on vascular responses [255]—this could be a useful future experimental avenue.

### 4.2. Systemic Applications of Capsaicin

In the recent relevant literature, the tissue-specific and systemic side effects of capsaicin have been rigorously studied. Despite its lipophilicity, local capsaicin administration does not result in any systemic bioavailability, a fact correlating with its poor aqueous solubility properties [256]. Here, it must be remarked again that systemic capsaicin administration correlates with a number of dose-dependent side effects [257]. Since most of these effects are usually GIT-related, they can now be mostly obviated by employing liposomal carriers, which release capsaicin directly into the blood flow [258,259]. In Table 3, based on selected scientific publications, the most notable systemic effects of capsaicin are presented.

**Table 3:** Systemic effects of capsaicin.

Indication	Formulation	Effect	Action Mechanism	Type of Study	Year	References
Systemic anti-inflammatory effect of somatostatin	Μice: 30, 60 and 90 mg/kg on 3 consecutive days under anaesthesia; guinea pigs: 2% capsaicin solution perineural for 30 min	Anti-inflammatory	Somatostatin release	In vivo—animal	2000	[260]
Burning mouth syndrome	3 capsules of capsaicin (50 mg of powder of red pepper with 0.25% capsaicin) a day for 1 month	Antalgic	Presumed inhibition of substance P	In vivo—human	2003	[261]
Possible protection against cancer, atherosclerosis and age-related diseases	10 μM	Antioxidant	Decrease in malondialdehyde level and protein carbonyl group content	In vitro—erythrocytes	2006	[262]
Helicobacter pylori gastritis	10 μg/mL	Anti-inflammatory	Inhibition of *H. pylori*-induced IL-8 production	In vitro—AGS or MKN45 cells	2007	[263]
Endometriosis	1M solution	Inhibition of proliferation of endometriotic cells	Inhibition of NF-kB	In vitro-immortalized stromal-like and epithelial-like endometriotic cells	2008	[264]
Irritable bowel syndrome	Pills 0.50 mg of capsaicin, 4 pills per day, 6 weeks	Antalgic, antibloating	Desensitisation of nociceptive receptors, depletion of substance P	In vivo—human	2011	[265]
Cardiovascular and metabolic diseases	1% red pepper powder which contains approximately 2.45 mg/g of capsaicin	Antilipidaemic, antiobesity	TRPV1 activation	In vivo—animal	2012	[266]
Chronic unexplained cough triggered by environmental irritants	1 capsule with 0.4 mg pure capsaicin 2 times/day, for 2 weeks, followed by 2 capsules with 0.4 mg pure capsaicin 2 times/day for 2 weeks	Antitussive	Desensitisation of the cough-sensitive TRPV1	In vivo—human	2015	[267]
Atherosclerosis	10, 20, 30, 40, and 50 μM	Antioxidant	Caspase-3 mediated pathways suppression	In vitro—macrophage RAW 264.7 cells	2015	[268]
Anoxia/Reoxygenation injury	10 μM to 40 μM	Cardioprotective	Upregulation of SIRT1 pathway	In vitro—rat cardiomyocytes	2017	[269]
Heart failure post myocardial infarction	0.1% cream, 150 μL/25 g	Cardioprotective	Induction of nociceptor-induced conditioning	In vivo—animal	2019	[270]
Hyperlipidaemia, oxidative stress, atherosclerosis	2.5, 5 and 10 mg/kg administered by gavage once daily	Anti-inflammatory, antioxidant, cardioprotective	Decreased total and LDL cholesterol, triglycerides, and apo B-100, and increased HDL cholesterol and SOD	In vivo—animal	2019	[271]
Hepatic steatosis	Cream 0.075%—8 week duration	Antilipidemic, antioxidative	Inhibition of β-oxidation, inhibition of hepatic lipogenesis	In vivo—animal	2020	[272]
Renovascular hypertension	0.006% capsaicin diet for 6 weeks	Antihypertensive	Increased phosphorylation of protein kinase B and endothelial NO synthase	In vivo—animal	2020	[273]
Acute inflammatory demyelinating polyneuropathy (AIDP)/Guillain-Barré syndrome (GBS) and chronic inflammatory demyelinating polyneuropathy (CIDP)	10 μM	Antioxidative, immunomodulatory	Reduction of IFN gamma-induced MHC-II production and decreased TLR4 and ICAM-1 mRNA expression	In vitro—Schwann cells	2020	[274]
Pentylenetetrazole-Induced Seizures	1 or 2 mg/kg	Anticonvulsant, neuroprotective	reduced glutathione (GSH), nitric oxide, and paraoxonase-1 (PON-1)	In vivo—animal	2020	[275]
Hypercholesterolemia	200 µM	Hypolipidemic	Upregulation of LDLR and downregulation of PCSK9 expression	In vitro-HepG2 cells	2022	[276]

In addition to all the aforementioned, in a recent experimental study, it was shown that capsaicin inhibits a series of proteins associated with the Warburg effect in sepsis and also downregulates cyclo-oxygenase 2 (COX-2) in a TRPV-1-independent manner [277]. This is important for a number of reasons; to start with, the Warburg effect, originally proposed in the 1920s [278,279], is essential for the metabolism of cancer cells [280], and its inhibition might provide an avenue for novel therapeutic strategies [268,281,282]. Secondly, the inhibition of COX-2, which is already the target of a number of drugs (e.g., [283,284,285]), means that capsaicin can be used in conjunction with them to enhance their effect. Finally, in the presence of TRPV-1 agonists other than capsaicin (e.g., [286,287,288]) or antagonists (e.g., [289,290,291,292]), this approach will, theoretically, still be functional.

### 4.3. Capsaicin as an Anti-Cancer Agent

The anticarcinogenic effect of capsaicin is mainly associated with the activation of TRPV1, which can be considered a probable link between inflammatory, immune and carcinogenic processes, as seen in Table 4.

**Figure 2:**
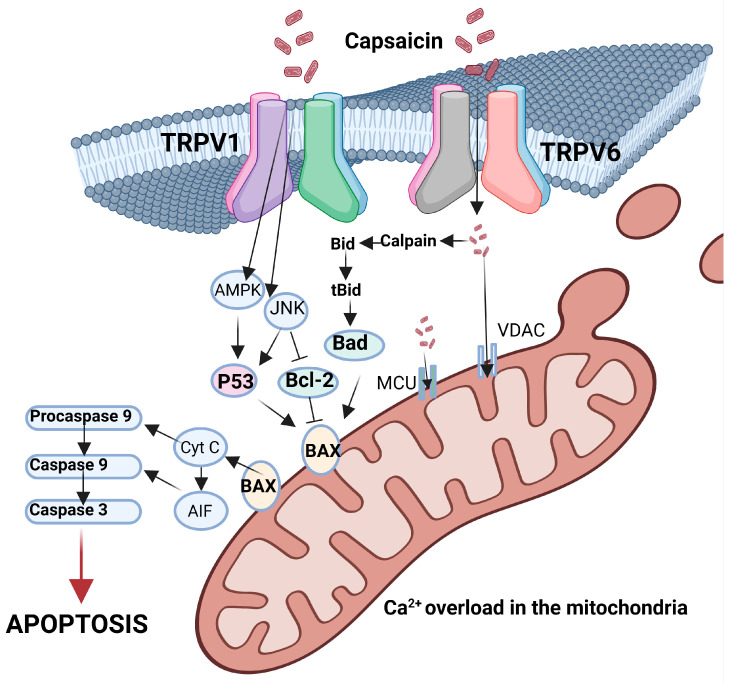
Apoptotic network triggered by capsaicin via TRPV 1 and TRPV6.

There are several events in the anti-cancer trajectory of capsaicin that were documented: antimutagenic activity, anti-oxidative action, anti-inflammatory action, cell cycle regulation and clear involvement in cancer cell death [328]. Out of all the mentioned molecular events associated with capsaicin’s anti-cancer action, the induction of cancer cell death is the most important, as capsaicin acts on multiple targets. As outlined in Figure 2, besides TRPV1, another member of the TRPV family involved in the anti-cancer action of capsaicin is TRPV6. Comparable to TRPV1, TRPV6 regulates calcium homeostasis. In in vitro studies, it was shown that capsaicin increases TRPV6 expression and increased levels of intracellular calcium ions that activate the calpain pathway for apoptosis [329]. Moreover, TRPV6 overexpression increased mitochondria permeability through the activation of Bax and p53 through C-jun N-terminal kinase (JNK) activation. Apoptosis can thus be induced by capsaicin in a TRPV1-dependent and independent manner. In the TRPV1 independent pathway, capsaicin activates adenosine 5-monophosphate-activated protein kinase (AMPK), p53 and JNK. When capsaicin binds to the mitochondrial complex I and II in the electron transport chain, the mitochondrial membrane potential is disrupted, and the membrane permeability is increased. Capsaicin increases ROS levels and increases the expression of pro-apoptotic Bcl-2 (Bax), as it was found in the case of neuroendocrine melanoma, a very aggressive and fatal tumour by Jun et al. [294,330,331]. This decreases the anti-apoptotic Bcl-2 and CytC release and induces apoptosis [317].

Some other anticarcinogenic applications of capsaicin should be mentioned here. It is possible to use capsaicin as a radio-sensitising agent in patients with prostate cancer; this particular use takes advantage of capsaicin-induced inhibition of NFκB signalling [332], resulting in angiogenesis inhibition [333]. More generally, recent studies explore the potential of combining capsaicin with conventional chemotherapeutic agents [334,335,336,337]. Other carcinogenesis-related signalling pathways may represent potential targets for future studies [338].

Another aspect we should consider is the increase of serum somatostatin induced by systemic capsaicin administration, which has already been noted by Thán et al. [260] and Szolcsányi et al. [339]. The release of somatostatin is associated with anti-inflammatory [340] and anti-nociceptive effects [341] in rats. The research of [342] has also focused on the somatostatin-induced inhibition of inflammation and nociception.

It is known that somatostatin is linked with such effects in humans [342,343], and somatostatin and its analogues have already been explored as targets for anti-cancer therapies [344,345,346,347,348,349,350,351,352,353]. The use of capsaicin in such a manner appears to be a promising avenue in cancer therapy research—a particular application could be in the case of hepatocellular cell carcinoma (HCC) where somatostatin and capsaicin application could be, in theory, effectively combined—the application of capsaicin in the pathogenesis of HCC specifically is explored by Scheau et al. [124].

The anticarcinogenic activity of capsaicin has also been a subject of in vivo studies, where chronic exposure to capsaicin seems to actually promote neoplasia by increasing collagen and elastin deposition [354] and by inhibiting NK cell function [355]. Capsaicin also exhibits a carcinogenic potential when combined with 9, 10-dimethylbenz[a]anthracene/12-O-tetradecanoylphorbol-13-acetate [356]. Finally, long-term capsaicin consumption favours metastasis because it modifies the microbiome of the gut, thus promoting the translocation of bacteria to the liver and altering bile acid metabolism, which ultimately inhibits NK cell function [357]; therefore, it must be examined in detail if and under which circumstances, the use of capsaicin may actually have detrimental effects in human health.

## 5. Capsaicin in Traditional Medicine Therapies

Originally, the capsaicin-containing plants of the genus Capsicum were native to Central and South America [358,359]. However, after the discovery of the Americas in the 16th century, it was quickly exported, as already mentioned, and gradually became a staple of many different culinary traditions [360].

While this genus comprises about 25 species, only five of them have been domesticated [361,362,363] and are commonly cultivated [364]; although the species is typically a perennial plant, it can be cultivated as an annual crop in areas with low temperatures [365,366]. Chile peppers, along with a number of other parts, were integral in the Mesoamerican civilisation’s agriculture [367] and even later in the formation of traditional Mexican cuisine [368]—the same has happened in a number of other localities, such as Pueblo in Colorado [369].

The traditional medical and even culinary usage of chilli peppers, and therefore capsaicin, is quite diverse [370]. While the domestication of the plant is estimated to have taken place somewhen before the 5th millennium BC [366], it may be assumed that they were also consumed sometime before [371] since the agriculture of many pre-colonial communities was pretty advanced ([372]; and references therein); the domestication process seems to have begun independently in a number of different areas [358]. Its significance is readily apparent from archaeological finds of the pre-Ceramic (ca. 9500–900 BC) and Formative (900 BC–250 AD) periods in South America [373,374] (time frames based on Lanning [375]). The millennia of chilli consumption must have given rise to a number of medicinal applications. In addition, a number of different civilisations that occupied pre-Columbian America, such as the Incas [376], Mayas [377], and Aztecs [378], used chilli peppers as war-related artefacts and for ritualistic purposes [6]. While these last two uses of chilli may be seen as atypical, on the one hand, it must be remarked that the absence of a monetary economy led to natural goods and materials having a more prominent role, a typical example being that of obsidian and other rocks and minerals [379,380]; on the other, a significant number of civilisations have used plants in ritualistic purposes [381,382,383].

It is known that capsaicin content differs between different Capsicum species [384]. Different foodstuffs also have, as expected, differing capsaicin contents [385], and this presumably influences their various uses to some extent. In fact, it is even possible to conceive the use of chilli peppers as a food-medicine continuum in the minds of the locals [386,387]. Perhaps the most diverse uses are recorded in Mexico, where chile is native, as is seen in Table 5; interestingly, the increased capsaicin consumption in parts of Mexico seems to correlate positively with adiposity and fat markers [388]. The complete spectrum of the local ethnobotanical use of chilli peppers is provided in a recent study [387]; various uses of different parts of the chilli plants are provided by Meghvansi et al. [389]. Miscellaneous or unverified uses of peppers, and thus capsaicin, also exist, such as those reported by Saleh et al. [390].

In addition, chilli is used along with other herbs and plants for a number of diseases or ailments related to the metaphysical concept of soul and evil energy [397,403,406,409,410,411]. It is possible that a number of medical applications of chilli peppers in traditional medical practice, especially for Native Americans, have been lost to time or have not yet been discovered. It must not be forgotten that Inca medicine, for example, was relatively advanced and possibly superior to contemporary European practises in some fields like surgery [412,413], as evidenced by a variety of findings and mentions in Spanish chronicles [414]. It is, therefore, entirely possible that a number of useful and effective applications of chilli extracts, as well as those of other plants, existed. In order to elucidate the full extent of the intertwining of food, medicine and culture in a local and traditional context, further research and novel practices are required [415,416].

On another note, we would like to point out that, as presented in the tables of the previous sections, based on previous research [63,64,65], capsaicin cream was applied to acupuncture point P6 or K-D2, which is the Korean equivalent, and also in LI4 [197] and ST36 [194]. Most, if not all, of the effects in these cases, are associated with some form of inhibition of the synthesis, transport and/or action of substance P; indeed, substance P is integral in the modern interpretation of the action of acupuncture in many pain states [417]. Traditional Chinese Medicine (TCM) is one of the most widely used traditional medicine systems in the world, and although it does not incorporate capsaicin-containing plants in its original, ancient phytochemical tradition [418,419], it is interesting to note this, apparently, as of yet, successful combination with capsaicin.

## 6. Side Effects of Capsaicin 

Extensive research has revealed a variety of physiological and pathological effects of capsaicin (Table 6); most but not all of capsaicin’s side effects are exerted by the activation of TRPV1. When applied locally, at the level of the skin or other external mucous membranes, it will induce skin erythema, neurogenic inflammation [420], non-blistering associated burning [421], marked lacrimation, blepharospasm and even conjunctivitis [422]. It must be noted here that a specific form of contact dermatitis, the so-called “Hunan hand” was first diagnosed in individuals who handled peppers daily due to their occupation. This is considered a clear and reliable marker of dermal capsaicin toxicity [423,424].

At the level of the CNS, capsaicin toxicity is associated with convulsions, excitement [425,426], disorientation and fear [427]; a host of other generalised symptoms, such as loss of body motor control, including diminished hand-eye coordination, have been reported [427].

In the cardiovascular system, capsaicin causes blood pressure increase and heart rate increase, and, in highly toxic levels, these effects may progress respectively to hypertension and tachycardia, with even ventricular fibrillation having been reported [427]. The blood pressure increase is associated both with the heart rate elevation and with the increased vascular contractility [428], leading to vasoconstriction. A summary of all the hypotheses and determined effects and side effects of capsaicin in the cardiovascular system in different modes of application has been provided in recent research [111]. Particular features of the cardiovascular system might predispose to or aggravate these responses [429,430,431,432].

At the level of the respiratory system, it causes bronchoconstriction and coughing [433], while in increased doses, it may even cause oedema of the larynx and the lungs, chemical pneumonitis and even respiratory arrest [434]; these data for capsaicin toxicity are derived from in vitro experiments with capsaicin analogues [434]. Systemic capsaicin toxicity has also been associated with pulmonary oedema and hyperventilation. A particular mechanism of neurogenic toxicity may be beneficial in controlling the neurogenic inflammation associated with nasal polyps, at least in some cases [182].

At the level of the gastrointestinal tract, an increased dose of capsaicin causes a general irritation, ranging from a local warmth sensation to a painful burning sensation [435]. It is also known that capsaicin influences gastric activity [436]. Despite capsaicin having some gastroprotective effects, it also has the potential to induce ulcers [275,437].

**Table 6:** Pathological effects in cases of capsaicin toxicity per body system.

System	TRPV1-Bearing Cell Types	Toxic Side-Effects	References
CNS	Cerebral neurons, sensory neurons of the dorsal root ganglion	Convulsions, excitement, disorientation, fear, loss of body motor control	[109,425,426,427]
Cardiovascular	Vascular smooth muscle cells, endothelial cells	Heart rate increase, blood pressure increase, hypertension, tachycardia, ventricular fibrillation, increased vascular contractility, atherosclerosis	[427,428]
Respiratory	Airway epithelial cells, T cells of the upper and lower airways	Bronchoconstriction, coughing, laryngeal oedema, pulmonary oedema, chemical pneumonitis, respiratory arrest	[114,433,434]
Gastrointestinal	Submucosal nerve plexus, myenteric nerve plexus, gastrointestinal mucosal cells, parietal and antral G cells	General irritation and pain, increased ulcer incidence	[117,275,435,437]
Integumentary	Unmyelinated type C and thin myelinated A_δ_ sensory nerve fibres, keratinocytes, mast cells, dermal blood vessels, fibroblasts, hair follicles, vascular smooth muscle cells, sebocytes and eccrine sweat glands	Skin erythema, non-blistering associated burning, “Hunan hand” (capsaicin-specific contact dermatitis)	[118,119,120,421,423,424]
Eyes	Corneal cells, retinal ganglion cells	Marked lacrimation, blepharospasm, conjunctivitis	[121,122,422]

From a medical standpoint, in cases of capsaicin overexposure, common adverse effects are painful skin reactions and systemic effects, like nausea, vomiting, abdominal pain and diarrhoea accompanied by a burning sensation [438]; capsaicin is toxic in far lower doses in children compared to adults. In the case of eye exposure, following contact with pepper sprays, marked lacrimation, pain, conjunctivitis, and blepharospasm are common and may be aggravated by the presence of risk factors [439,440]. For local toxic reactions, a thorough decontamination of the skin and mucous membranes is recommended [441], involving water and antiacids [442]; furthermore, the treatment of systemic toxicity is based on the management of symptoms until capsaicin excretion [443].

Finally, a few fringe cases of capsaicin toxicity are reported in the medical literature, namely an acute polyneuropathy, presenting as Guillain-Barre syndrome following pepper spray exposure [444], the death of an infant after a capsaicin-containing traditional medicine was orally administered [445], and an acute MI in a patient with a transdermal capsaicin patch [446].

## 7. Discussion and Future Research Perspectives

Currently, as a phytomedical compound, capsaicin has been demonstrated to have analgesic, antioxidant, anti-inflammatory, anti-cancer, cardio-protective, and metabolic modulation effects. Capsaicin analogues are also currently evaluated for such properties [447]. A recent study even documented capsaicin-induced inhibition of cell senescence [448], while another proposed that capsaicin may even be a viable management option in cases of schizophrenia [449]; regarding the cardio-protective effects of capsaicin, it might even be possible to use it to alleviate acute myocardial injury [450]. Considering that the majority of such mechanisms are caused by the activation and subsequent inactivation of the TRPV1 receptor, further studies of the role of this receptor may yield useful results regarding both diagnostic and treatment methods. Notably, TRPV1 belongs to a category of receptors recently characterised as extra-oral taste receptors, i.e., oral receptors not found in the oral cavity [451,452]. Outside of the oral cavity, these receptors appear to have immune system-related and bronchorelaxation-associated properties [453,454]; it is known that taste receptors and their associated effector biomolecules are expressed in tuft-1 cells [455,456,457,458]. The characteristic morphology of these cells has been described in detail by Hendel et al. [459]; their localisation is quite diverse [455,460,461]. Moreover, it seems that they are also involved in the regulation of the immune system [462].

A significant challenge of using capsaicin for its potent therapeutic properties is its poor bioavailability due to its quick metabolisation [463]. It is believed that in vivo capsaicin concentrations achieved through conventional routes of administration are inferior to the levels that demonstrated effectiveness in vitro [464]. This is due to variations and limitations in absorption, distribution, and excretion, which limit the permeation of capsaicin to the desired action site. Therefore, in vivo, replication of the effects observed in vitro is an increasing focus of interest, and effective methods are being researched in this regard [465,466,467]. Furthermore, systemic capsaicin administration is associated with a number of side effects, and so in order to produce the maximum possible therapeutic effect in the target tissue while, at the same time, minimising side effects, it is desirable to control its delivery with precision. This can be performed, as previously alluded to, by employing novel delivery systems, namely liposomes, micelles, micro-emulsions and nano-emulsions [468,469], colloidal capsules and solid nanoparticles [470]; another avenue concerning implant-associated infections [471] would be the integration of capsaicin into 3D printed biomaterials [472]. These improve oral bioavailability for targeted applications, including anti-cancer endeavours [473,474]. Combination of capsaicin with bioenhancing substances such as piperine can prevent its degradation and increase its systemic concentration [475,476]. A number of nanostructured lipid carriers can also be incorporated into transdermal patches to reduce local side effects, such as skin irritation and erythema [477]—the use of capsaicin in the management and treatment of skin pathologies is a promising and rapidly developing field [118]. A future perspective on increasing capsaicin concentration for anti-cancer effects is also its integration into a delivery system that responds to physiologic triggers such as temperature or local pH, therefore optimising its clinical use and expanding its potential as an anti-cancer therapeutic agent [156,478,479]. Capsaicin, as well as other phytochemicals with promising medicinal properties [480], may benefit from such novel delivery methods. A novel way for capsaicin delivery for a particular case of colorectal cancer has recently been explored by Rajput et al. [481]. 

In cases of inflammation, either local or systemic, capsaicin may offer a good alternative if the common anti-inflammatory drugs are not tolerated due to their side effects. The combination of capsaicin with acupuncture may also be useful in that regard, given that acupuncture is already quite effective in the treatment of pain (e.g., [482,483,484,485]) and other inflammatory states (e.g., [486,487,488]), and the relevant research interest is increasing [489].

Of particular interest is the emerging research on the antibacterial (e.g., [490,491,492]), antifungal (e.g., [493,494]), antiviral (e.g., [495]) and antiparasitic (e.g., [496]) properties of capsaicin. Already, a number of different phytomedical compounds and their derivatives are being researched for their antimicrobial/antiviral potential, such as kaempferol [497,498,499,500], quercetin [501,502,503], curcumin [504,505,506,507], coumarin [508,509,510], and allicin [511,512,513]. This is especially important when considering the increasing antimicrobial resistance (e.g., [514,515,516,517,518,519,520]) and the occasional severe side-effects like allergies to antimicrobial drugs (e.g., antibiotics [521,522]) and especially some antiparasitic drugs ([523] and references therein). 

Furthermore, other research directions could involve capsaicin’s role in modulating intestinal microbiota, of whose diversity it increases; this opens new possibilities in combating the complications of various GIT-related illnesses [524,525] through the modulation of the gut-brain axis and immune system interaction [526]. Potential applications of capsaicin in pulmonary and gastrointestinal cancers have also been concisely summarised in recent papers [527,528]. An interesting research direction could also involve the application of capsaicin at acupuncture points, aside from the aforementioned combination of capsaicin and acupuncture; indeed, based on the positive results of three clinical trials [63,64,65] where capsaicin was applied locally at acupuncture points, larger-scale research for a number of conditions can be undertaken, following the same principle—similar positive research results were also reported by Kim et al. [194,197]. When considering the proposed special properties of meridians in interstitial fluid—or generally fluid—circulation [529,530,531,532], it is compelling to consider the potential for applying specialised cutaneous treatment schemes, using capsaicin or even other bioactive compounds, in this manner.

## 8. Conclusions

Capsaicin is a potent phytochemical substance that has numerous health benefits. It can be used medicinally both in systemic and local administration. At the same time, the potential toxicity of capsaicin poses an important constraint on its medicinal use, especially in certain sensitive tissues such as the eyes. Already, capsaicin forms part of a number of medical traditions, and such proposed medicinal uses warrant further research. The association between capsaicin and acupuncture must also be explored more thoroughly. Based on the data presented in this paper, we conclude that capsaicin may be used as a monotherapy or adjunct therapy in the treatment or management of a number of pathologies.

## Figures and Tables

**Table 1:** General and medical uses of capsaicin.

Use of Capsaicin and Its Derivatives		References
Animal repellents		[43,44,45,46]
Food industry—fragrance ingredient		[47,48,49]
Pesticides		[50,51,52,53]
Veterinary medicine (various uses)		[54,55,56]
Medical Uses	Chronic pain—cream application (local adm.)	[57,58,59,60,61]
	Gastroprotection in cases of drug administration	[62]
	Post-operative nausea and vomiting	[63,64,65]
	Post-operative sore throat	[66]
	Pruritus	[67,68,69,70,71]
	Urinary bladder hyperactivity	[72,73]
	Skin conditioning creams	[74,75]

**Table 4:** Anti-cancer effects of capsaicin.

Indication	Formulation	Effect	Action Mechanism	Type of Study	Year	References
Hepatocellular carcinoma	200 mM capsaicin or Met-capsaicin	Pro-apoptotic, potential chemopreventive	DNA fragmentation/nuclear condensation/activation of caspase-3	In vitro—SK-Hep-1 hepatocellular carcinoma cells	2001	[131]
Colon cancer	200–300 mM	Proapoptotic, chemopreventive	PPARγ pathway activation (non-TRPV1 related)	In vitro—HT-29 human colon cancer cells	2004	[133]
Gastric adenocarcinoma	1 mmol/L	Proapoptotic	Reduction of Bcl-2 and antiapoptotic protein/DNA fragmentation	In vitro AGS cells	2005	[134]
Prostate cancer	Different doses	Profound antiproliferative effect	Prevention of NF-kappaB activation	In vitro—prostate cancer cell lines/In vivo—prostate cancer cell xenografts on mice	2006	[135]
Gastric cancer	50 μM	Proapoptotic	TRPV6-mediated capsaicin-induced apoptosis/stabilization of p53 through JNK-regulated p53 phosphorylation/increase of Bax and p53 protein without increasing transcription	In vitro—human gastric cancer AGS cells/GES-1 cells	2007	[293]
Melanoma	50–200 μM	Proapoptotic	Down-regulation of Bcl-2 expression, nuclear condensation, internucleosomal DNA fragmentation	In vitro—B16-F10 melanoma cells	2007	[294]
Benzo(a)pyrene induced experimental Lung cancer	10 mg/kg	Chemoprotective	Decreased lung mitochondrial lipid peroxidation	In vivo—animal	2008	[295]
Pancreatic cancer	2.5 mg capsaicin/kg body weight 5 times/week, 5 mg capsaicin/kg 3 times/week by oral gavage (in vivo part)	Proapoptotic	Increased expression of Bax, down-regulation of Bcl-2, significant release of cytochrome c and AIF in the cytosol	In vivo—athymic nude mice; in vitro—AsPC-1 and BxPC-3 cells	2008	[296]
Small cell lung cancer	Oral administration of 10 mg capsaicin/kg of mice; 50 Μm capsaicin for cell cultures	Antiproliferative	Decreased expression of E2F-responsive proliferative genes cyclin E, thymidylate synthase, cdc25A and cdc6at	In vivo–animal, in vitro-human SCLC cell lines NCI-H69, NCI-H82, DMS53 and DMS114	2010	[297]
Chronic Pancreatitis and pancreatic intraepithelial neoplasia	Oral administration of 10 ppm. capsaicin or 20 ppm depending on research group	Chemopreventive	Reduction of cell proliferation and suppressed phosphorylation of ERK and c-Jun, blocked Hedgehog/GLI pathway activation	In vivo—animal	2011	[298]
Breast cancer	200 μM	Proapoptotic	Decreased mitochondria membrane potential, induced cleavage of PARP-1, decreased procaspase-7 expression	In vitro—MCF-7 and BT-20 human breast cancer cell lines	2011	[299]
Tongue cancer	50, 100 and 150 µM	Proapoptotic, prooxidant	Activation of caspase 3, DNA fragmentation, induction of G_0_/G_1_ phase arrest, activation of ROS	In vitro—SCC-4 human tongue cancer cells	2012	[300]
Colorectal cancer	50 and 100 μM	Anti-proliferative, proapoptotic	Suppression of TCF-4 expression and disruption of TCF-4 and β-catenin interaction	In vitro—SW480, LoVo, and HCT-116 colorectal cancer cells	2012	[301]
Bladder cancer	50, 100, 150, 200 µM	Anti-proliferative, proapoptotic	Inhibition of CDK2, CDK4 and CDK6; cell death induction by ROS increase and decreased mitochondrial membrane potential	In vitro—5637 human urinary bladder cancer cell line	2012	[302]
Human KB cancer cells	1, 50, 100, 150, 200 and 250 μM	Anti-proliferative, proapoptotic	Mitochondrial membrane permeabilization and caspase activation	In vitro—human KB cancer cells	2013	[303]
Gastric cancer	10–300 μM	Anti-proliferative (probably), proapoptotic	Decreased expression of phosphorylated ERK 1/2, p38 MAPK or JNK	In vitro—human gastric cancer cells (AGS cells)	2014	[304]
Cholangiocarcinoma	150–200 µM	Impaired cell proliferation, migration, invasion, epithelial to mesenchymal transition growth inhibition in soft agar colonies	Inhibition of Hedgehog signalling pathway	In vitro—human CC cell lines (SZ-1 and TFK-1)	2014	[305]
Pancreatic neuroendocrine tumours	10–200 μM	Cytotoxic	Disruption of mitochondrial membrane potential and inhibition of ATP synthesis	In vitro—BON and QGP-1 cells	2014	[306]
Bladder cancer	10–250 μM	Mediation of cancer cell apoptosis	Activation of dendritic cells via CD91	In vitro—T24 and SD48 human urinary bladder cancercell lines	2015	[307]
Prostate cancer	50, 100, 150 and 200 μM	Antiproliferative	Restoration of miR-449a profiling in cancer cells leading to negative modulation of the androgen receptor	In vitro—human C4-2 and LNCaP cells	2015	[308]
Bladder cancer	100 and 200 μM	Antiproliferative, anti-migration, cell cycle prolongation	Inhibition of tNOX and sirtuin 1 (SIRT1)	In vitro—TSGH8301 and T24 urinary bladder cancer cells	2016	[309]
Gastric cancer	0–16 μg/mL	Chemopreventive, antiprofirative, proapoptotic	Reduced hMOF activity	In vitro—colon cancer SW-480, gastric cancer MGC-803 and gastric mucosal GES-1 cells	2016	[310]
Prostate cancer	20, 80 μM	Antiproliferative, induced autophagy	Activation of ROS generation, increased levels of LC3-II, accumulation of p62	In vitro—prostate cancer (LNCaP and PC-3) cells	2016	[311]
Renal cell carcinoma	0–400 μM	Proapoptotic	Up-regulation of pro-apoptotic genes (c-myc, FADD, Bax andcleaved-caspase-3,-8, and-9), down-regulation of Bcl2, activated p38 and JNK MAPK pathways	In vitro—human renal cell carcinoma 786-O, ACHN, Caki-1 cells	2016	[312]
Ovarian cancers	0.1–50 μg/mL	Proapoptotic	Cell cycle arrest	In vitro—SKOV-3 ovarian cancer cells/in vivo-male SD rats	2017	[313]
Nasopharyngeal carcinoma	100, 150, 200 and 300 μM/L	Antiproliferative, proapoptotic, induced autophagy	Increased G_1_ phase cell cycle arrest, increased LC3-II and Atg5 levels, decreased p62 and Fap-1 expression, increased caspase-3 activity	In vitro—NPC-TW01 cells	2017	[314]
Oesophageal squamous cell carcinoma	120 µM	Antiproliferative, propapoptotic	Inhibition of glycolysis, decreased HK-2 expression	In vitro—Het-1A cell	2018	[315]
Oral squamous cancer	50–350 µM	Antiproliferative, proapoptotic	Disruption of the mitochondrial-membrane potential, activation of caspase-3, -7 and -9, DNA fragmentation	In vitro—ORL-48 cells	2019	[316]
Osteosarcoma	20 µM	Proapoptotic	Mitochondrial dysfunction, overproduction of ROS and JNK, activation of AMPK-p53 pathway	In vitro—MG63 cells	2019	[317]
Breast cancer	0, 10, 50, 100 or 200 µM	Proapoptotic	Induced G_2_/M cell cycle arrest, reduced CDK8 expression levels, decreased phosphorylation of PI3K and Akt, downregulation of Wnt and β-catenin	In vitro—MDA MB 231 breast cancer cells	2020	[318]
Prostate cancer	1, 5, 10 µM	Proapoptotic	Decreased expression of Wnt-2, p-GSK3β, β-catenin, c-myc and cyclin D1	In vitro—PC-3 and DU145 prostate cancer stem cells	2020	[319]
Gastric cancer	IC_50_ of 0.6 ± 0.0421 μM	Antiproliferative	Inhibition of histone methylation KDM1A	In vitro—gastric cancer cell line BGC-823	2020	[320]
Glioblastoma	(IC_50_) values of capsaicin were 325.7 ± 12.4 μM at 24 h and 265.7 ± 10.2 μM at 48 h	Proapoptotic	Upregulation of peroxisome proliferator-activated receptor gamma	In vitro—human glioblastoma LN-18 cell line	2020	[321]
Breast cancer	In vitro: 150 μΜ/L for 72 h; in vivo: 10 mg/kg 1 time per 3 days for 21 days	Proapoptotic, antiproliferative	Down-regulation of FBI-1-mediated NF-κB pathway	In vivo—female BALB/c athymic nude mice; in vitro—human breast cancer cell lines (MCF-7 and MDA-MB-23)	2021	[322]
Lung cancer cells	0–200 µM	Antiproliferative	Reduced accumulation of HIF-1α protein inhibition of mitochondrial respiration	In vitro—A549, H1299, H2009, and H23 cell lines	2022	[323]
Renal cancer	0, 5, 10, 25, 50, 100 and 200 μM	Inhibition of cell migration, invasion and epithelial-mesenchymal transition Induced autophagy	AMPK/mTOR pathway	In vitro—renal cell carcinoma (RCC) 786-O and CAKI-1 cell lines	2022	[324]
Epithelial lung cancer NSCLC	100, 200 and 300 μM/L	Inhibition of proliferation and promotion of ferroptosis	Increase of total iron levels and ferrous ion levels by regulating the SLC7A11/GPX4 axis	In vitro—NSCLC cells (A549 and NCI-H23)	2022	[325]
Hepatocarcinogenesis	100, 200 and 300 μM/L	Significant inhibition of hepatocarcinogenesis	Inhibition of SIRT1/SOX2 signalling; SIRT1 downregulation	In vitro—HepG2 and WB-F344 cells	2022	[326]
Anaplastic thyroid cancer	50, 100 and 200 μM	Stemness-inhibitory effect	Calcium-dependent autophagic degradation of OCT4A, following TRPV1 activation	In vitro—8505C and FRO cells	2022	[327]

**Table 5:** Some traditional medical applications of capsaicin.

Use	Tribe(s)/Country	Capsicum Species Used	References
Antibacterial/antimicrobial	Pimas/Mexico	*Capsicum* sp.	[391]
Nematicide	Pimas/Mexico	*Capsicum* sp.	[392,393]
Fever alleviation	Zapotecs and Raramuris/Mexico	*Capsicum* sp.	[394,395]
Mental, behavioural and neurological disorders	Raramuris and Mestizos/Mexico	*C. annuum* var. *glabriusculum*	[395]
Ocular pathologies treatment	Mestizos and Zapotecs/Mexico	*C. annuum* var. glabriusculum	[394,396,397,398,399]
Auricular pathologies treatment	Nahua/Mexico	*C. annuum* var. glabriusculum	[394,398,399]
Respiratory pathologies treatment	Nahua and Mestizos/Mexico	*Capsicum* sp.	[77,397,400,401,402,403]
Pathologies of the gastrointestinal tract	Mestizos, Nahua and Lacandon Maya/Mexico	*Capsicum* sp.	[77,397,400,401,404,405]
Skin pathologies	Mestizos, Zapotecs and Tzotzil/Mexico	*Capsicum* sp.	[77,396,397,403,406,407]
Disease of the musculoskeletal system and associated tissues	Ramamuris/Mexico	*Capsicum* sp.	[395,408]
Pathologies of the genito-urinary system	Local tribes/Mexico	*Capsicum* sp.	[77,400,401]
Labor pain mitigation/delivery promotion	Local tribes/Mexico	*Capsicum* sp.	[400]
Poisoning treatment (for spider bites)	Mestizos/Mexico	*C. annuum* var. *glabriusculum*	[400,403]
General health benefits	Local people/Eritrea	*Capsicum* sp.	[390]

## Data Availability

No new data were created or analyzed in this study. Data sharing is not applicable to this article.
